# Development of a More Environmentally Friendly Silk Fibroin Scaffold for Soft Tissue Applications

**DOI:** 10.3390/jfb14040230

**Published:** 2023-04-18

**Authors:** Nathan V. Roblin, Megan K. DeBari, Sandra L. Shefter, Erica Iizuka, Rosalyn D. Abbott

**Affiliations:** 1 Department of Materials Science and Engineering, Carnegie Mellon University, Pittsburgh, PA 15213, USA; nroblin@andrew.cmu.edu (N.V.R.);; 2 Department of Biomedical Engineering, Carnegie Mellon University, Pittsburgh, PA 15213, USA; 3 Department of Mechanical Engineering, Carnegie Mellon University, Pittsburgh, PA 15213, USA

**Keywords:** biomaterials, tissue engineering, silk fibroin, sodium carbonate, sodium hydroxide, environmental impact, green processing, soft tissue, adipose tissue, adipocyte

## Abstract

A push for environmentally friendly approaches to biomaterials fabrication has emerged from growing conservational concerns in recent years. Different stages in silk fibroin scaffold production, including sodium carbonate (Na_2_CO_3_)-based degumming and 1,1,1,3,3,3-hexafluoro-2-propanol (HFIP)-based fabrication, have drawn attention for their associated environmental concerns. Environmentally friendly alternatives have been proposed for each processing stage; however, an integrated green fibroin scaffold approach has not been characterized or used for soft tissue applications. Here, we show that the combination of sodium hydroxide (NaOH) as a substitute degumming agent with the popular “aqueous-based” alternative silk fibroin gelation method yields fibroin scaffolds with comparable properties to traditional Na_2_CO_3_-degummed aqueous-based scaffolds. The more environmentally friendly scaffolds were found to have comparable protein structure, morphology, compressive modulus, and degradation kinetics, with increased porosity and cell seeding density relative to traditional scaffolds. Human adipose-derived stem cells showed high viability after three days of culture while seeded in each scaffold type, with uniform cell attachment to pore walls. Adipocytes from human whole adipose tissue seeded into scaffolds were found to have similar levels of lipolytic and metabolic function between conditions, in addition to a healthy unilocular morphology. Results indicate that our more environmentally friendly methodology for silk scaffold production is a viable alternative and well suited to soft tissue applications.

## 1. Introduction

In recent years, growing environmental concerns have led to a push for the development of environmentally friendly approaches for materials processing and fabrication. These alternative approaches typically include efforts to reduce energy consumption and to reduce the use of hazardous and polluting processing methods. In some cases, particularly in the biomaterials field, this can also include efforts to move away from materials with potentially harmful effects on human populations in favor of safer options.

Silk fibroin sourced from the domesticated mulberry silkworm (*Bombyx mori*) can be processed into a wide variety of formats, allowing it to be used in a number of regenerative applications ranging from wound dressings to tissue engineering of adipose depots, bones, cartilage, and ligaments [1], as well as applications in other fields including textiles and cosmetics. Silk fibroin has several advantages that make it well suited to a plethora of biomedical applications. The mechanical properties of fibroin can be tuned by controlling the crystalline structure of the fibroin protein, specifically the β-sheet content [2,3,4,5]. Fibroin also has a highly tunable degradation rate that depends on processing conditions, resulting in a wide range of degradation profiles with non-cytotoxic byproducts [6,7,8,9,10]. Fibers taken post-degumming can either be woven directly into sutures [4] or dissolved in ionic liquids for further processing. Aqueous fibroin can be used to fabricate thin films, hydrogels, microspheres, or 3D microporous scaffolds, among other formats [10].

While silk can be sourced from a variety of insects and arachnids—notably silkworm and spider species—*Bombyx mori* is one of the most commonly used and extensively characterized sources of silk for biomedical applications. *Bombyx mori* silk in particular has been used in a wide range of applications due to the relative cost-effectiveness of processing, ease of harvesting, and increased availability compared to spider silk spidroin, as well as the challenges associated with spider farming [11,12].

Unlike other silk sources, silkworm cocoons are primarily composed of two proteins: fibroin, the fiber protein that comprises most of the cocoon and provides structural support, and the sericin protein family, the glue-like coating that binds fibers together [2,5,10,13,14,15]. In isolation, silk fibroin is biocompatible and produces minimal inflammatory response and immunogenic effects in the body [16,17,18,19,20,21]. However, composites of fibroin in combination with sericin can produce an inflammatory response [2,5], and thus, separation of the two proteins is a key step in silk biomaterials fabrication.

The established protocol for processing *Bombyx mori* cocoons to fabricate silk fibroin biomaterials takes advantage of sodium carbonate (Na_2_CO_3_) and lithium bromide (LiBr) for the degumming and solubilization stages of silk processing, respectively [10]. This easy-to-use methodology has been widely accepted and is ubiquitous in current research involving silk fibroin biomaterials, largely owing to its ability to yield consistent fibroin solutions for biomaterials manufacturing [13]. However, sodium carbonate is currently produced via the Solvay process, which typically relies on ammonia non-sustainably sourced from processes with high levels of electricity consumption and greenhouse gas emissions [22]. The Solvay process also releases large amounts of solid calcium chloride waste products, resulting in high levels of pollution [23]. Even discounting non-sustainable material sources, silk degumming via sodium carbonate requires the use of a hot plate, which has extremely low heating and energy efficiencies, as well as large volumes of water [10,24]. To avoid the detrimental environmental impacts of sodium carbonate degumming, some promising alternatives for silk processing have been proposed that would be more environmentally friendly than the established methodology [25]. Unlike sodium carbonate, previous studies have demonstrated that sodium hydroxide (NaOH) is capable of degumming *Bombyx mori* silk fibroin at room temperature, without the need for a heating element [13,26]. Sodium hydroxide also drastically reduces water usage requirements during the silk processing stages and can be sustainably sourced from salt brines [13,26,27]. However, the use of NaOH as a degumming agent has not been optimized and was previously paired with other non-green processes (i.e., HFIP-based processing [13,26,27]). Furthermore, the resulting silk biomaterials processed using NaOH have not been widely studied, characterized, or used in soft tissue applications.

Among various silk biomaterial formats, silk fibroin scaffolds are of particular interest for their ability to provide a support matrix for a wide variety of tissues in regenerative applications. The two most used methods for manufacturing microporous scaffolds are the HFIP-based method and the “aqueous-based” method [10,28]. In the HFIP method, lyophilized (freeze-dried) silk fibroin is redissolved in the corrosive, volatile, and highly toxic organic solvent 1,1,1,3,3,3-hexafluoro-2-propanol (HFIP) and then methanol-annealed to form β-sheets, whereas in the aqueous-based method, aqueous silk fibroin is exposed to NaCl salt to undergo dehydration-induced β-sheeting [10,25,29]. In both methods, NaCl or a different porogen (i.e., gas bubbles) is used during processing to create a controlled pore architecture within the scaffolds, with tunable pore size in proportion to porogen diameter [10,28]. Studies have shown that aqueous-based fibroin scaffolds have increased surface roughness [28] and have more rapid degradation [30] relative to scaffolds produced via the HFIP method. However, the environmental impact of the aqueous-based method is drastically reduced by eliminating the need for HFIP and methanol [25], and the aqueous-based approach is a faster method for scaffold production [10]. Despite encouraging early results supporting the use of aqueous-based silk scaffolds for soft tissue applications [31], much of the research to date using silk biomaterials has focused on HFIP-based scaffolds [31,32,33,34,35,36,37,38,39,40,41,42,43,44].

The primary aim of this research was to develop a more “green” approach for silk fibroin scaffold production that replaces some key processing steps with more environmentally friendly alternatives, while yielding scaffolds with comparable properties to those fabricated via established non-sustainable methods. This was performed by optimizing an NaOH protocol to replace Na_2_CO_3_ as a silk degumming agent, and then combining this with the established aqueous-based methodology for scaffold fabrication. Degummed silk fibers resulting from the experimental NaOH degumming process were compared against Na_2_CO_3_-degummed silk fibroin, with compositional, morphological, and gravimetric analyses to assess relative sericin removal. Aqueous-based scaffolds produced after either degumming step were then characterized to compare scaffold morphology, composition, volumetric porosity, wet compressive modulus, and in vitro proteolytic degradation. Human adipose-derived stem cells were seeded in NaOH- and Na_2_CO_3_-degummed aqueous-based silk scaffolds to compare cell viability and attachment. Lastly, scaffolds using both degumming methods were seeded with human whole adipose tissue to compare tissue adhesion and morphology, as well as lipolytic function and metabolic activity of adherent adipocytes.

## 2. Materials and Methods

### 2.1. Iterative Development of Optimized NaOH Degumming Methodology

A multi-stage experiment was carried out to determine an optimal protocol for degumming *Bombyx mori* silk cocoons using sodium hydroxide (NaOH), where the goal was to yield similar results to control samples degummed using sodium carbonate (Na_2_CO_3_) [10]. In the prior literature, a few methods for using NaOH to separate *Bombyx mori* silk proteins have been attempted, with only limited characterization of the resulting silk fibroin. Methodology varied significantly between studies, with NaOH concentrations ranging from 0.025 M to 0.5 M, utilizing continuous stir or shaking, and degumming times of up to 24 h [13,26]. As a first step toward optimization, degumming time and technique were fixed at 24 h of continuous stirring—the longest and most rigorous conditions reported in prior work—while the concentration of the NaOH degumming agent was varied at intervals from 0.025 M up to 0.5 M. The degumming ratios for NaOH-degummed fibers from each NaOH degumming concentration were compared against control samples. Simultaneously, SEM micrographs of silk fibers after degumming were imported into FIJI 2.9.0 software, and measured fiber diameters were compared. Three images were taken per degummed fiber sample, and at least 25 randomly chosen fibers were measured from each image (*n* ≥ 375).

**Scheme 1:**
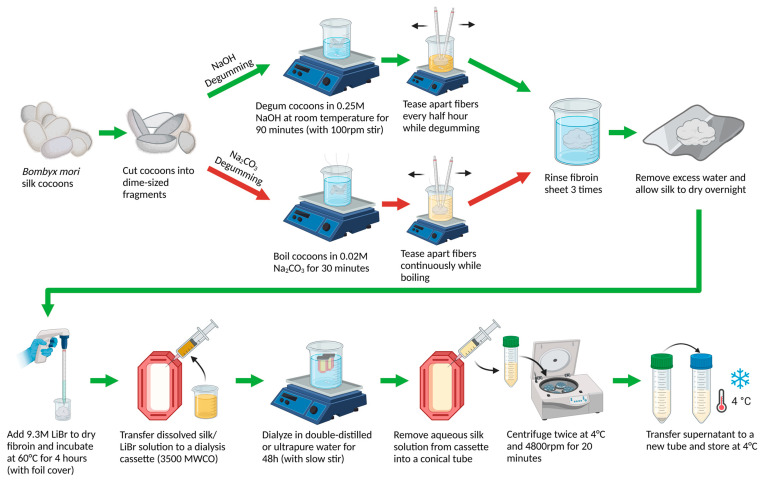
Processing steps to produce aqueous fibroin solutions using silkworm cocoons, with either the standard degumming method (red arrows) [10] or an experimental “green” degumming technique. Created with Biorender.com.

The next stage of the experiment focused on degumming duration. Modified from the established Na_2_CO_3_-based method [10], a protocol (explained in more detail in Scheme 1 below) was developed that involved 90 min degumming using the concentration determined to be optimal (0.25 M NaOH), while intermittently teasing apart fibers manually. This was compared against the control samples, along with the already optimized 24 h degumming method from the previous stage at the same NaOH concentration. Similar comparisons of degumming ratio and fiber diameter were used to evaluate the new modified degumming protocol.

### 2.2. Preparation of Aqueous Silk Fibroin

#### 2.2.1. Degumming *Bombyx mori* Cocoons

Silk fibroin was extracted from *Bombyx mori* cocoons using either an established method [10] or the optimized experimental degumming protocol (Scheme 1).

Control group silk fibroin samples were extracted using the established degumming protocol [10]. Whole cocoons (OliverTwistsFibres, Durham, UK) were each cut into four dime-sized pieces and weighed out to 2 g, before boiling in 0.02 M sodium carbonate (Sigma–Aldrich, St. Louis, MO, USA) solution for 30 min at a 400:1 liquor ratio (400 mL solution per 1 g silk) while continuously teasing apart fibers manually. Silk fibers that remained after degumming were rinsed in double-distilled water to remove sericin and sodium carbonate residues and left to dry overnight at room temperature.

The experimental degumming protocol was developed to extract silk fibroin samples from *Bombyx mori* cocoons using sodium hydroxide (NaOH) at a 50:1 liquor ratio (50 mL solution per 1 g silk), modified from the established protocol [10]. An amount of 2 g of cocoon pieces were prepared according to the established protocol, and sodium hydroxide pellets (Sigma–Aldrich, St. Louis, MO, USA) were used to prepare 100 mL of a 0.25 M NaOH solution. The NaOH solution was transferred to a beaker with a stir bar, and cocoon fragments were degummed for 90 min at room temperature with continuous 100 rpm stir, and fibers were teased apart for 5 min every half hour during the process. Silk fibers that remained after degumming were rinsed in double-distilled water to remove sericin and NaOH residues and left to dry overnight on a clean paper towel at room temperature.

For each degumming condition, the initial dry mass of cocoon fragments (approximately 2 g) was recorded as W_1_, and the mass of degummed silk after drying was recorded as W_2_. The degumming ratio (η), a gravimetric analysis of removed mass, was then calculated as a percentage using the following equation:

η = (1 − (W_2_/W_1_)) × 100%. (1)

#### 2.2.2. Dissolution of Silk Fibroin

Dry silk fibroin was dissolved following an established protocol [10]. A 9.3 M lithium bromide (Sigma–Aldrich, St. Louis, MO, USA) solution was added to dry fibroin, which was subsequently incubated at 60 °C for 4 h. This solution was then removed into 3500 MWCO dialysis cassettes (Thermo Fisher Scientific, Waltham, MA, USA) for 48 h dialysis using double-distilled water with slow continuous stirring. The water was replaced six times during dialysis. The remaining solution was then centrifuged twice at 4 °C and 4200 rpm for 20 min, and the purified supernatant fibroin solution was saved and stored at 4 °C.

### 2.3. Preparation of Silk Scaffolds

Aqueous-based scaffolds were produced following an established protocol [10]. Aqueous silk solutions were diluted to 6 wt% silk fibroin and 3 mL was aliquoted to LDPE sample containers (Kartell Labware, Noviglio, MI, Italy). NaCl (Sigma–Aldrich, St. Louis, MO, USA) was sifted to isolate crystals with diameters of 500–600 µm, and 6 g was added slowly and uniformly into each container. Containers were tapped down gently to remove air bubbles. Containers were capped and left at room temperature for two days to allow silk fibroin to gel via dehydration-induced β-sheet formation [29]. Fibroin sponges were then soaked in double-distilled water for 48 h with repeated water changes to leach the salt porogen. Sponges were then cut into cylindrical scaffolds with 2 mm height and 4 mm diameter before being dried for storage. Scaffolds were autoclaved in distilled water prior to use in sterile applications.

### 2.4. Characterization of Silk

#### 2.4.1. Scanning Electron Microscopy

Degummed fibers and silk scaffolds were imaged using an FEI Quanta 600 FEG SEM and TESCAN MIRA 3 SEM at Carnegie Mellon University’s Materials Characterization Facility. Degummed fibers and scaffolds were coated with a 5 nm layer of gold particles using a sputter coater prior to imaging, since fibroin is not naturally conductive. Images were taken using backscattered electrons at an accelerating voltage of 30 kV. Scaffolds were scanned at low magnitudes (30×) to show morphology and pore structure (*n* = 15), whereas fibers were scanned at higher magnifications (80× and 320×) to distinguish individual fibers for diameter measurements (*n* = 375) and to show any unremoved sericin residue (*n* = 15). Gold sputter coating and sample mounting on carbon tape rendered SEM a destructive endpoint for scaffolds.

#### 2.4.2. Attenuated Total Reflection (ATR) Spectroscopy

Crystallinity and composition analyses of silk fibroin at different stages of processing were determined by analyzing absorbance spectra produced from Fourier-Transform Infrared-Attenuated Total Reflection spectroscopy (FTIR-ATR). Measurements were made using a PerkinElmer Frontier IR/NIR system, with 32 accumulation scans per measured sample across the wavenumber range of 600–4000 cm^−1^, and then baseline-corrected in post-processing [45]. Due to the pressure required to secure samples for scanning, FTIR-ATR was considered a destructive endpoint. Crystallinity of silk fibers (*n* = 15) and scaffolds (*n* = 15) was determined by a comparison of scanned amide I and amide II peaks to their characteristic silk I and silk II wavenumber ranges (Table 1).

Determination of relative sericin content in primarily fibroin fibers post-degumming was determined through comparison of absorbance intensity ratios (I_1650_/I_1625_, I_1400_/I_1445_, and I_1070_/I_1165_) at wavenumbers that are indicative of characteristic differences between sericin and fibroin bonding peaks (Table 2) [45].

#### 2.4.3. Scaffold Porosity Determination

Scaffolds were dried overnight after cutting to specified dimensions, and the dry mass was subsequently recorded. Scaffolds were then impregnated with hexane by submerging in hexane and applying vacuum for 5 min before repressurization. Hexane-impregnated scaffolds were then weighed in a sealed conical tube to prevent evaporation during measurement. Porosity was calculated (*n* = 15) using known densities of silk fibroin (1.348 g/mL) and hexane (0.659 g/mL):

(2) $$\varepsilon \left(\%\right)=\frac{W_{2}-W_{1}}{\rho_{hex}}/\left[\frac{W_{2}-W_{1}}{\rho_{hex}}+\frac{W_{1}}{\rho_{silk}}\right]\times 100\%$$

#### 2.4.4. Wet Compressive Testing

Compression testing was performed on silk scaffolds (*n* = 15) using an MTS Criterion system with a 50 N load cell. Tests were conducted at room temperature on wet samples (pre-soaked for 16 h in double-distilled water), with compression up to 90% strain at a strain rate of 0.02 mm/s. Compressive stress–strain curves were plotted, and wet compressive moduli of scaffolds were calculated from the initial linear portion of the curve.

#### 2.4.5. Degradation via Proteolytic Enzyme

Silk scaffolds (*n* = 15) were exposed to protease XIV from *Streptomyces griseus* (Sigma–Aldrich, St. Louis, MO, USA) to monitor and compare enzymatic degradation profiles over the course of 21 days, as an in vitro model of scaffold degradation [47,48,49]. After recording the initial scaffold mass dry, scaffolds were plated in a 48-well plate and each submerged in 500 µL of 181 µg/mL (1 U/mL) protease XIV solution (prepared in sterile distilled water, with approximately 2% penicillin/streptomycin), with continuous incubation at 37 °C. The protease solution was changed daily for each scaffold. On three-day intervals, scaffolds were taken out of enzyme solution and rinsed 3 times in sterile distilled water before drying (8 h at 60 °C) to monitor changes in dry mass across measurement timepoints. Changes in dry mass for each scaffold were normalized to initial scaffold mass to compare percent reduction over the course of the study. Discarded protease solution was denatured by incubation for 20 min at 80 °C according to the manufacturer’s protocol.

### 2.5. Determination of Cellular Responses to Silk Scaffolds

Following scaffold characterizations, separate sets of aqueous-based silk scaffolds prepared via both previously described degumming methods were used to assess interactions with various adipose cell types, particularly human adipose-derived stem cells (hASCs) and whole adipose tissue. The first set of scaffolds was used to assess hASC viability three days post-seeding in aqueous-based silk scaffolds. Further assessments were performed on separate aqueous-based silk scaffolds three days after seeding with whole adipose tissue from human patients. A small subset of scaffolds was formalin-fixed, stained for nuclei, lipids, and F-actin, and subsequently imaged via confocal microscopy. After completing formalin-fixed sample imaging, other assays were performed on another subset of scaffolds to assess DNA content, lipid content, and lipolytic function (via secreted glycerol) from scaffolds. Lastly, fluorometric metabolic assays were performed on extra unused scaffolds to confirm the viability of cells in seeded tissue.

#### 2.5.1. Seeding Scaffolds with Human Adipose-Derived Stem Cells (hASCs)

Human adipose tissue was received from an abdominal panniculectomy (patient: 40-year-old female with a BMI of 22, non-diabetic) conducted at the University of Pittsburgh Medical Center (UPMC). Human adipose-derived stem cells (hASCs) were isolated from human adipose tissue as described previously [32] and frozen for long-term storage. The hASCs were later thawed and seeded into cell culture. Cells were expanded in cell culture media (DMEM, high glucose, 10% FBS, 1% penicillin/streptomycin) (Thermo Fisher Scientific, Waltham, MA, USA) to achieve sufficient populations for seeding into scaffolds.

Silk scaffolds with uniform dimensions were autoclaved in distilled water for sterilization, and then submerged in cell culture media overnight for protein adsorption to allow for cell attachment to fibroin. Scaffolds were each seeded with 500,000 hASCs (250,000 cells per side) lifted from culture flasks at passage 2, and immediately placed in 37 °C incubation. After a 2 h incubation period (to allow for cell attachment after seeding), fresh cell culture media was added to scaffolds for continued incubation. Seeded scaffolds were cultured for three days.

#### 2.5.2. Live–Dead Staining for Viability Determination

Fluorescent viability assays were performed (*n* = 15) on day three post-seeding using an Invitrogen LIVE/DEAD Viability/Cytotoxicity kit (Thermo Fisher Scientific, Waltham, MA, USA) as indicated, with membrane-permeable calcein-AM to stain living hASCs green and membrane-impermeable ethidium homodimer-1 to stain dead hASC nuclei red. Z-stack scans were taken immediately after staining, using a Zeiss LSM 700 confocal microscope with 488 nm and 555 nm lasers. Maximum intensity projections were created using FIJI 2.9.0 software, and then live and dead cell counts were performed to determine percent viability in each scaffold.

#### 2.5.3. Seeding Scaffolds with Whole Adipose Tissue

Liquefied adipose tissue was received from procedures (first patient: liposuction of a 36-year-old male with a BMI of 25; second patient: liposuction of a 70-year-old female with a BMI of 32; third patient: abdominal panniculectomy of a 54-year-old female with a BMI of 35; fourth patient: abdominal panniculectomy of a 28-year-old female with a BMI of 30; all non-diabetic) conducted at the University of Pittsburgh Medical Center (UPMC). To remove blood, lipoaspirate was washed 3 times with an equal volume of sterile DPBS (Thermo Fisher Scientific, Waltham, MA, USA) prior to use.

Autoclaved silk scaffolds with uniform dimensions were submerged in cell culture media (DMEM, high glucose, 10% FBS, 1% penicillin/streptomycin) overnight for protein adsorption to allow for cell attachment to fibroin. Scaffolds were submerged in lipoaspirate and placed in 37 °C incubation for 1 h to allow for cellular infiltration as described previously [42,44]. Seeded scaffolds were then removed from lipoaspirate, placed into a 24-well plate, and incubated at 37 °C. After a 2 h incubation period, cell culture media was added to scaffolds for continued incubation. Seeded scaffolds were cultured for three days.

#### 2.5.4. PicoGreen Assay

A subset of scaffolds (*n* = 15) was removed from the media on day three post-seeding, and each scaffold was placed in 500 µL 1X TE buffer (Thermo Fisher Scientific, Waltham, MA, USA) for storage at –20 °C. Scaffolds were re-thawed and dissected in TE buffer immediately prior to use, and Invitrogen Quant-iT PicoGreen assay kits (Thermo Fisher Scientific, Waltham, MA, USA) were used following the manufacturer’s protocol to assess the DNA content of adherent adipose tissue in scaffolds. The data were also used to normalize subsequent assays for each scaffold to its total DNA content.

#### 2.5.5. Triglyceride Assay

Immediately after completing the PicoGreen assays, EnzyChrom Triglyceride assays (BioAssay Systems, Hayward, CA, USA) were performed on the same subset of re-thawed scaffold samples (taken on day three post-seeding) following the manufacturer’s protocol. Data for the triglyceride content from adherent adipocytes (*n* = 15) were normalized to paired PicoGreen data to account for any differences in cell density.

#### 2.5.6. Glycerol Assay

On day three post-seeding, the surrounding media from the same subset of scaffolds that were used for the PicoGreen/Triglyceride assays was removed and stored at −20 °C. Following the manufacturer’s protocol, EnzyChrom Glycerol assays (BioAssay Systems, Hayward, CA, USA) were performed on re-thawed media to assess lipolysis from adherent adipose tissue in scaffolds via relative glycerol secretion into the media. The data from each media sample (*n* = 15) were normalized to paired PicoGreen data for the corresponding scaffold to account for any differences in cell density.

#### 2.5.7. Fixed Tissue Staining in Silk Scaffolds

A separate subset of scaffolds (*n* = 4) was formalin-fixed on day three post-seeding, and fluorescently stained with DAPI (Sigma–Aldrich, St. Louis, MO, USA), BODIPY FL (Thermo Fisher Scientific, Waltham, MA, USA), and AlexaFluor 555 Phalloidin (Thermo Fisher Scientific, Waltham, MA, USA) to localize the nucleus, lipids, and F-actin, respectively. Z-stack scans were taken after staining, using a Zeiss LSM 700 confocal microscope with 405 nm, 488 nm, and 555 nm lasers. Maximum intensity projection maps were created from Z-stacks using FIJI 2.9.0 software. Silk fibroin autofluorescence across multiple excitation wavelengths allowed for the scaffold pore walls to be distinguished from stain targets. Images were used for non-quantitative morphological assessment only.

#### 2.5.8. Resazurin Assay

After completing formalin-fixed sample imaging and other assays, fluorometric resazurin metabolic assays were performed on extra unused scaffolds (*n* = 10). Resazurin (Thermo Fisher Scientific, Waltham, MA, USA) was diluted to a stock concentration of 1 mM in PBS, and subsequently diluted down to a working concentration of 0.05 mM in cell culture media (DMEM, high glucose, 10% FBS, 1% penicillin/streptomycin) [39]. Warmed resazurin-in-media solution was introduced to seeded scaffolds on day three of culture as a replacement for cell culture media, and then incubated at 37 °C for 2 h. In total, 10 scaffolds of each type were used for metabolic analysis. Fluorescence measurements (Ex/Em = 560/590 nm) were taken according to the manufacturer’s protocol.

### 2.6. Statistics

Experiments with degummed silk fibers were each performed using at least five independently prepared silk batches per condition. Experiments with aqueous-based silk scaffolds were each performed using scaffolds taken from three independently prepared silk batches per condition, with the exception of the fluorometric resazurin metabolic assay and fixed-tissue staining (which each used scaffolds from only two independently prepared silk batches per condition). Statistics were performed with GraphPad Prism 9 software (GraphPad, San Diego, CA, USA). An ordinary one-way analysis of variance (ANOVA) was used to determine statistical significance for the degumming ratio and fiber diameter between each degumming condition during optimization. Unpaired *t*-tests were used for all later statistical comparisons that included only two conditions. Multiple unpaired *t*-tests were used to analyze degradation via proteolytic enzyme, with individual *t*-tests performed per timepoint. Statistically significant differences were always defined as *p* ≤ 0.05 and represented by a single asterisk (*) on plots. Further degrees of statistical significance were represented by additional asterisks (with 2–4 asterisks indicating *p* ≤ 0.01, *p* ≤ 0.001, and *p* ≤ 0.0001, respectively). Comparisons that were not found to be statistically different (*p* > 0.05) were represented on plots as “ns” (not significant).

## 3. Results and Discussion

The goals of this paper were to (1) optimize a green alternative degumming approach using NaOH, (2) characterize the NaOH degummed fibers against the gold standard degumming process [10], (3) combine the optimized green degumming step with aqueous-based scaffold production to develop a more environmentally friendly scaffold, (4) compare environmentally friendly scaffolds to the established control across a wide array of characterizations, and (5) verify that the newly developed scaffold protocol is biocompatible with hASCs and whole adipose tissue.

### 3.1. Iterative Optimization of NaOH Degumming Methodology

**Figure 1:**
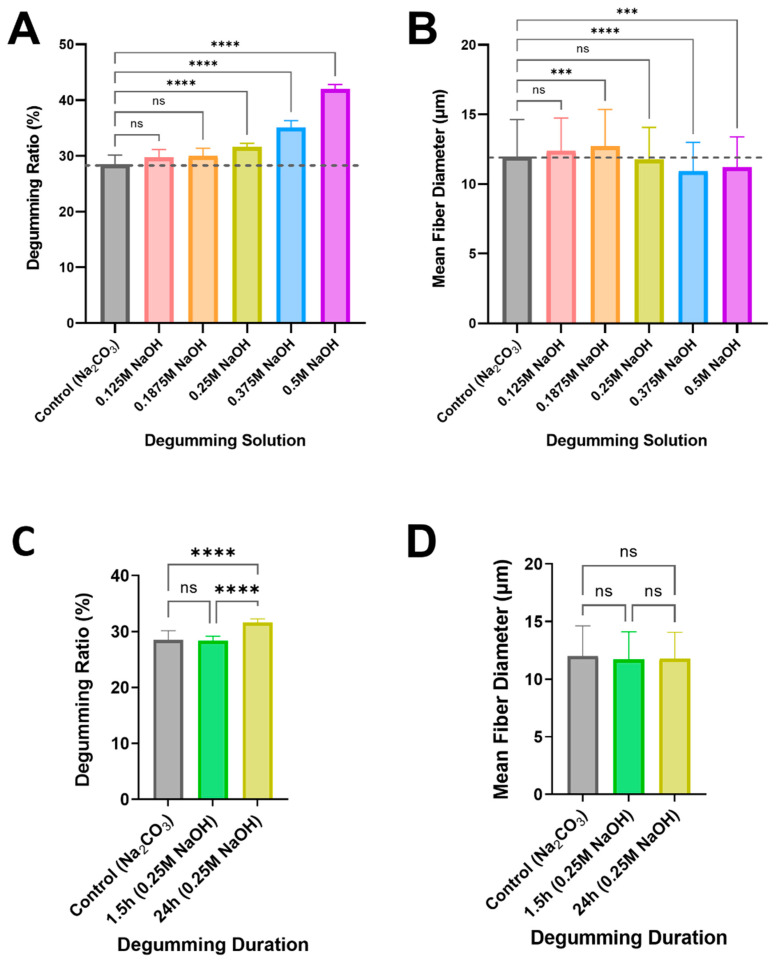
Degumming with 0.25 M NaOH for 90 min was found to be optimal for matching both sericin removal and fiber diameter yield from sodium carbonate degumming. Iterative development of a protocol for degumming *Bombyx mori* cocoons using NaOH, with control samples degummed using 0.02 M Na_2_CO_3_ following an established protocol [10]: (**A**,**B**) Experimental samples degummed using varied concentrations of NaOH for 24 h, using unpaired *t*-tests between the control and each experimental condition to compare (**A**) gravimetric degumming ratio (*n* ≥ 5), and (**B**) silk fiber diameters measured in FIJI 2.9.0 software (*n* ≥ 375). (**C**,**D**) Experimental samples degummed using 0.25 M NaOH for either 90 min or 24 h, using unpaired *t*-tests between each condition to compare (**C**) degumming ratio (*n* ≥ 5), and (**D**) silk fiber diameters measured in FIJI 2.9.0 software (*n* ≥ 375). Asterisks (*) indicate statistical significance (*p* ≤ 0.05) from unpaired *t*-tests comparing datasets within each panel, with additional asterisks representing a greater degree of statistical significance (*** indicates *p* ≤ 0.001, while **** indicates *p* ≤ 0.0001). Comparisons that were not found to be statistically different (*p* > 0.05) were represented on plots as “ns” (not significant).

Since the removal of sericin is key to avoiding detrimental immunogenic effects [16], we first wanted to optimize NaOH sericin removal. Preliminary results during the first stage of optimization indicated that 0.025 M NaOH was incapable of full sericin removal, as nearly intact cocoons were observed after degumming for 24 h. As a result, this condition was not continued for further replicates and analysis. For the remaining NaOH concentrations (0.125 M to 0.5 M), degumming ratios and measured fiber diameters from SEM micrographs were compared against the established Na_2_CO_3_-degummed control fibers. Each concentration of NaOH used for 24 h resulted in a degumming ratio higher than that of the control (Figure 1A), with significant increases for concentrations ≥ 0.25 M. Results indicate that components other than sericin were also being removed from the silk fibers, and thus that 24 h was too long of a duration for NaOH-based degumming. From fiber diameter measurements (Figure 1B), it was additionally determined that NaOH concentrations ≤ 0.1875 M were insufficient to fully remove the sericin coating from silk fibroin, as fiber diameters were larger than those in control samples (degummed with Na_2_CO_3_), while 0.25 M NaOH was capable of sufficiently removing sericin from silk fibers. As a result, 0.25 M NaOH was chosen for the next stage of the optimization process, in which it was combined with the modified 90 min degumming protocol that more closely resembled the established Na_2_CO_3_ degumming technique [10]. The degumming ratio for the 90 min degumming method at 0.25 M NaOH was not found to be significantly different from the control, unlike the 24 h degumming method at the same concentration of NaOH (Figure 1C). Additionally, fiber diameter was not found to significantly differ as a function of degumming duration at 0.25 M NaOH, or to the Na_2_CO_3_-degummed control (Figure 1D). Although the optimized NaOH degumming protocol utilized a longer degumming duration than the established Na_2_CO_3_-based method, a prior study demonstrated that degumming time does not influence β-sheet content of degummed silk or resulting scaffolds [50], and thus crystallinity for the optimized experimental process was expected to match the established control. Collectively, the results indicate that the 90 min degumming protocol using 0.25 M NaOH yielded similar results to the control degummed with Na_2_CO_3_, and thus was an optimal alternative degumming method.

### 3.2. Characterization of Degummed Silk Fibers

To determine whether the experimental NaOH-based degumming method would yield isolated fibroin that was compatible with scaffold manufacturing, it was imperative to first characterize experimentally degummed fibers for both sericin content and fibroin structure and compare results to the well-established Na_2_CO_3_ degumming process. Prior research demonstrated that degumming with the gold standard 0.02 M Na_2_CO_3_ for 30 min yields isolated fibroin in its silk II structure, with sericin sufficiently removed to avoid immunogenic effects [39].

**Figure 2:**
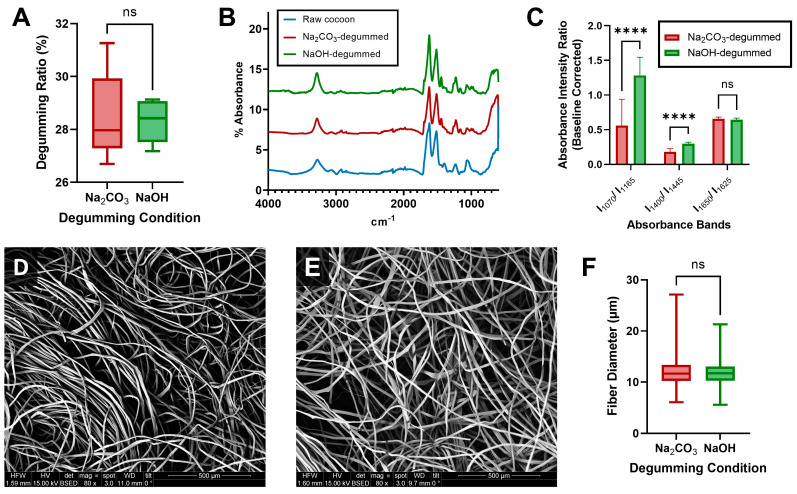
Sodium hydroxide degumming removes a similar percentage of sericin and results in the same isolated silk fibroin fiber diameter and morphology as sodium carbonate degumming. Degummed silk fibers were produced after using either Na_2_CO_3_ or NaOH as a degumming agent (*n* = 15). (**A**) Relative mass loss during the degumming process (represented as the degumming ratio), comparing cocoon mass to that of degummed fibers as a marker of sericin removal. (**B**) Representative FTIR-ATR spectra from degummed silk fibers showing absorbance peaks in the wavenumber range of 4000–600 cm^−1^, compared against an additional raw cocoon control to demonstrate baseline sericin and fibroin peaks before processing. (**C**) Baseline-corrected [45] absorbance ratios from three absorbance band pairs that are positively correlated to relative sericin content: 1070/1165 cm^−1^, 1400/1445 cm^−1^, and 1650/1625 cm^−1^. (**D**,**E**) Representative SEM micrographs of (**D**) Na_2_CO_3_-degummed silk fibers and (**E**) NaOH-degummed silk fibers. (**F**) Silk fiber diameters (*n* ≥ 375), measured in FIJI 2.9.0 software. Asterisks (*) indicate statistical significance (*p* ≤ 0.05) from unpaired *t*-tests comparing datasets within each panel, with additional asterisks representing a greater degree of statistical significance (**** indicates *p* ≤ 0.0001). Comparisons that were not found to be statistically different (*p* > 0.05) were represented on plots as “ns” (not significant).

Degumming ratio was again used as an initial assessment of relative sericin removal by each degumming method (Figure 2A). No significant differences were found between the two degumming methods, indicating similar amounts of sericin were removed. Furthermore, calculated ratios (~28 wt% removed) align with expected sericin content (25–30 wt%) in *Bombyx mori* cocoons.

After fibers were dried post-degumming, samples were taken for FTIR-ATR scans, analyzing amide I and amide II peaks to determine the secondary structure of isolated fibroin (Figure 2B). Relevant sericin-fibroin absorbance intensity ratios [45] were also examined to determine relative sericin content in silk fibers (Figure 2C). From ATR absorbance spectra, degummed fibroin was found to have amide I and amide II peaks at 1621 ± 3 cm^−1^ and 1513 ± 2 cm^−1^, respectively, regardless of the degumming method. Results indicate that fibroin isolated using either method was in its silk II (crystalline) conformation. Additionally, from Figure 2C, it can be seen that both methods resulted in I_1650_/I_1625_ and I_1400_/I_1445_ ratios closely matching the literature values for fully degummed fibroin [45], although NaOH-degummed fibers had a higher I_1400_/I_1445_ ratio compared to the Na_2_CO_3_-degummed control. This finding could indicate increased fibroin degradation by NaOH at alanine sites, which are a primary component of the hydrophobic repeat units found in silk fibroin [51,52]. Additionally, NaOH-degummed fibers had an elevated I_1070_/I_1165_ ratio compared to both Na_2_CO_3_-degummed fibers and the literature values [45]. While increases in each of these intensity ratios can suggest possible sericin residue, elevated I_1070_/I_1165_ ratio in particular has also been observed to be an effect of UV aging-related degradation of pure silk fibroin [45], and other degumming metrics indicated complete sericin removal. A possible explanation for this difference in I_1070_/I_1165_ ratio is the use of a stronger alkaline solution at a longer duration in the NaOH-degumming method, which could degrade fibroin into smaller constituents that contribute to underlying absorption peaks. Prior research with the established degumming agent has demonstrated that longer degumming times result in increased degradation of silk fibroin to smaller molecular weights [50], which would align with these findings. An alternate explanation is preferential degradation of fibroin at polar tyrosine sites compared to nonpolar amino acids (i.e., glycine and alanine), as this would decrease the absorbance at the 1165 cm^−1^ peak that tyrosine is primarily responsible for [45], and would explain the resulting increase in the I_1070_/I_1165_ ratio.

SEM imaging of degummed fibers confirmed that sericin was removed by each degumming method (Figure 2D,E), as scarcely any sericin (bright particle residue on fibroin fibers) could be observed on the imaged fibers. Additionally, Figure 2F indicated that despite the higher alkaline concentration and degumming time for the NaOH-degumming method, there was no statistically significant difference in fiber diameter between the methods. As fiber diameter has been shown to be a key predictor of certain mechanical properties (i.e., elastic modulus, ultimate tensile strength, and yield stress of fibers) [53], it is likely that the mechanical behavior of degummed fibers is similar between degumming methods, although further testing would be required to investigate this. Collectively, these results indicate that using a more environmentally friendly degumming process (sodium hydroxide) yields similar sericin removal and silk fibroin fiber morphologies to the current gold standard (sodium carbonate).

### 3.3. Characterization of Aqueous-Based Silk Scaffolds

**Figure 3:**
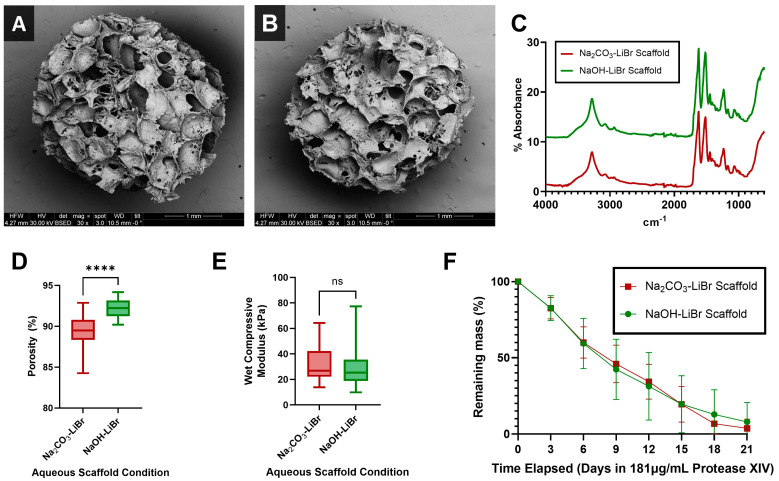
Aqueous-based silk scaffolds degummed with NaOH had higher porosity than Na_2_CO_3_-degummed scaffolds, but otherwise had similar morphologies, protein structure, mechanical properties, and degradation kinetics. Aqueous-based silk scaffolds produced following either Na_2_CO_3_- or NaOH-based degumming (*n* = 15). (**A**,**B**) Representative SEM micrographs of aqueous-based silk scaffolds prepared following (**A**) Na_2_CO_3_ degumming and (**B**) NaOH degumming. (**C**) Representative FTIR-ATR spectra from aqueous-based silk scaffolds showing absorbance peaks in the wavenumber range of 4000–600 cm^−1^. (**D**) Percent porosity by volume for aqueous-based silk scaffolds. (**E**) Wet compressive modulus (MPa) for aqueous-based silk scaffolds. (**F**) Percent of initial scaffold mass remaining after 37 °C incubation in 181 µg/mL protease XIV from *Streptomyces griseus*, with no significant differences found between conditions at any timepoint, assessed via unpaired *t*-tests for data at each timepoint. Asterisks (*) indicate statistical significance (*p* ≤ 0.05) from unpaired *t*-tests comparing datasets within each panel, with additional asterisks representing a greater degree of statistical significance (**** indicates *p* ≤ 0.0001). Comparisons that were not found to be statistically different (*p* > 0.05) were represented on plots as “ns” (not significant).

After producing aqueous fibroin solutions isolated via the two degumming methods, an environmentally friendly aqueous-based scaffold fabrication method was performed and the resulting scaffolds were analyzed for pore wall morphology (SEM), fibroin protein structure (FTIR-ATR), porosity (gravimetric analysis with hexane), wet compressive modulus, and degradation kinetics (via proteolytic enzyme). Scaffolds prepared using the two different degumming methods had similar pore wall morphologies (Figure 3A,B), with a mixture of large smooth pores formed from the NaCl salt porogen as well as smaller pores with observable surface roughness. This morphology is a known result of non-homogenous gelation, which occurs due to the relationship between aqueous fibroin concentration and NaCl porogen diameter in 3D microporous scaffold processing [28]. As expected, for both degumming methods, amide I and amide II absorbance peaks (Figure 3C) were found at 1620 ± 2 cm^−1^ and 1514 ± 1 cm^−1^, respectively, indicating the expected silk II structure and confirming β-sheet re-formation occurred during aqueous-based scaffold preparation. Interestingly, while both degumming methods resulted in high porosity, there was a significantly higher porosity in NaOH-degummed scaffolds (Figure 3D). 

In addition to composition and morphology, matching mechanical properties to the host tissue for a given application is a key consideration for scaffolds, as this will dictate the mechanical environment for cells and has implications for cell morphology, viability, proliferation, and differentiation [54,55,56,57]. In soft tissue applications, the primary loading mechanism to consider is compressive loading [58], and thus wet compressive modulus was an important characterization. Wet compressive moduli for scaffolds produced via the two degumming conditions were not significantly different from each other (Figure 3E). Calculated moduli were additionally found to be similar to that of soft tissues (i.e., adipose tissue: 2–25 kPa) [59], suggesting that pairing environmentally friendly sodium hydroxide degumming with aqueous-based processing methods provides an ideal matrix environment for soft tissue applications. Degradation kinetics are also a key consideration, as negative outcomes can result from tissue grafts when scaffolds persist beyond the initial phase of the wound healing process, including an inappropriate immune response, biofilm formation, and lack of host cell integration [30,60]. Over the course of 21 days in protease XIV from *Streptomyces griseus*, percent degradation was not significantly different between conditions at any timepoint (Figure 3F). Furthermore, the degradation of NaOH-degummed scaffolds was the same as Na_2_CO_3_-degummed scaffolds by the 21-day measurement, a physiologically relevant timescale for host tissue integration prior to remodeling [61,62]. Together, results from scaffold characterizations indicate that aqueous-based scaffolds produced using a more environmentally friendly degumming process (NaOH) have comparable morphology, protein composition, compressive modulus, and degradation kinetics to scaffolds prepared using the established Na_2_CO_3_ degumming method, while higher porosity could potentially provide better access to nutrients for cells in NaOH-degummed scaffolds.

### 3.4. Cellular Responses to Aqueous-Based Silk Scaffolds Degummed with NaOH and Na_2_CO_3_

**Figure 4:**
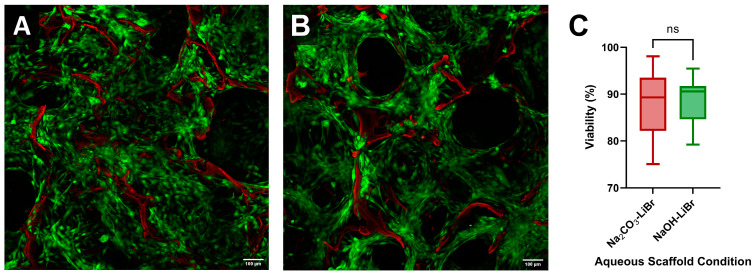
Aqueous-based silk scaffolds degummed with NaOH or Na_2_CO_3_ resulted in high hASC viability and uniform cell attachment to pore walls. Silk scaffolds seeded with tissue (*n* = 15). (**A**,**B**) Representative Z-stack max projection images of (**A**) Na_2_CO_3_-degummed and (**B**) NaOH-degummed silk scaffolds, seeded with hASCs and subsequently stained with calcein AM (green) and ethidium homodimer-1 (red) to indicate live and dead cells, respectively. Ethidium-stained silk (red) is also visible. (**C**) Percent viability for hASCs seeded in silk scaffolds, determined using live and dead cell counts. A comparison that was not found to be statistically different (*p* > 0.05), from an unpaired *t*-test comparing datasets, was represented on the plot as “ns” (not significant).

Silk scaffolds have been used for a variety of applications from soft tissues to bone [63]. Here we focused on soft tissue fabrication methods and developing a more environmentally friendly protocol. While our lab and others [31,32,33,34,35,36,37,38,39,40,41,42,43,44] have traditionally used sodium carbonate degumming with an HFIP solvent for adipose tissue engineering approaches, aqueous-based methods have shown similar results in vivo [31]. Since no prior studies have combined a sodium hydroxide degumming approach with the aqueous-based method, we next sought to determine whether cell attachment, morphology, and viability were affected by the fabrication protocol we developed. Confocal imaging (Figure 4A,B) demonstrated that hASCs were seeded uniformly into aqueous-based silk scaffolds, attaching primarily to available surfaces from scaffold pore walls with high viability for both scaffold conditions (Figure 4C). A slight non-significant increase in viability was observed on average for NaOH-degummed scaffolds, which is likely a result of their higher porosity allowing for better access to cell media nutrients.

**Figure 5:**
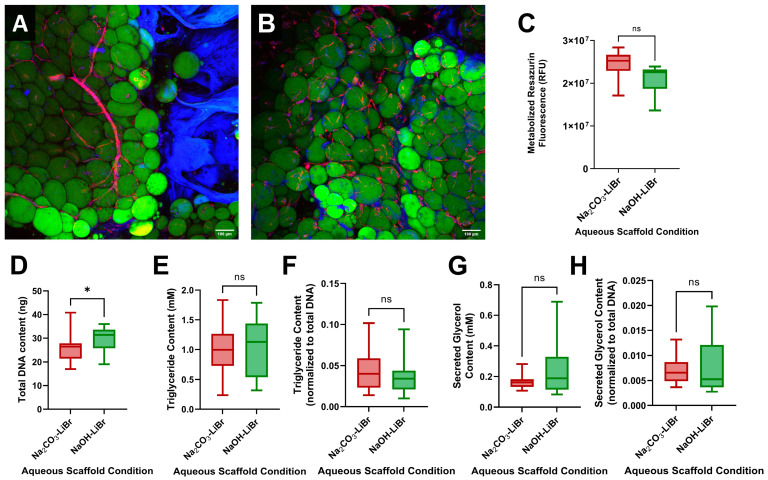
Aqueous-based silk scaffolds degummed with NaOH resulted in a higher degree of cell attachment, but otherwise adherent adipocytes exhibited the same healthy unilocular morphologies and lipid content between conditions, with similar lipolytic and metabolic function. Silk scaffolds seeded with human whole adipose tissue. (**A**,**B**) Representative Z-stack max projection images of (**A**) Na_2_CO_3_-degummed (*n* = 4) and (**B**) NaOH-degummed silk scaffolds (*n* = 4). Scaffolds were formalin-fixed and stained with DAPI (blue), BODIPY (green), and AlexaFluor 555 Phalloidin (red), with visible silk autofluorescence at similar excitation wavelength to DAPI (represented in blue) [64]. DAPI staining was used for visualization purposes only. BODIPY and phalloidin were used for the morphological assessment of adipocytes and SVF cell types, respectively. (**C**) A set of scaffolds (*n* = 10) were assessed for metabolic activity via fluorometric measurement for metabolized resazurin (RFU) from both Na_2_CO_3_- and NaOH-degummed aqueous-based silk scaffolds. (**D**–**H**) A set of scaffolds (*n* = 15) were assessed for (**D**) Total DNA content via PicoGreen assays, (**E**) triglyceride content (mM) via triglyceride assays, (**F**) triglyceride content normalized to total DNA per scaffold, (**G**) secreted glycerol content in surrounding media via glycerol assays, and (**H**) secreted glycerol content normalized to total DNA per scaffold. Asterisks (*) indicate statistical significance (*p* ≤ 0.05) from unpaired *t*-tests comparing datasets within each panel. Comparisons that were not found to be statistically different (*p* > 0.05) were represented on plots as “ns” (not significant).

Next, we evaluated whole adipose tissue seeded in the scaffolds. Seeded adipocytes displayed a healthy unilocular morphology (Figure 5A,B) with a corresponding high presence of stromal vascular fraction cells present (vascular endothelial cells and stem cells characterized by phalloidin staining), indicating that each of the primary components of whole adipose tissue were able to attach to the scaffolds. Measured fluorescence from resazurin-stained scaffolds (Figure 5C) indicated metabolism of the resazurin dye by seeded cells, and thus that viable cells are present in scaffolds of each condition three days after seeding with whole adipose tissue. No significant difference in fluorescence was determined between the two conditions. Total DNA content within the scaffolds (Figure 5D) indicated that aqueous-based silk scaffolds produced following NaOH degumming had a significant increase in DNA content. This is likely due to a higher degree of cellular attachment in the more porous NaOH-degummed scaffolds. To evaluate adipocyte functionality, triglyceride content (Figure 5E) and glycerol secretion (Figure 5G) were then determined for each scaffold and normalized per scaffold using total DNA content (Figure 5F,H, respectively). No significant differences were found in triglyceride content or glycerol secretion between conditions, before or after normalizing to the DNA content. This indicates similar lipid content and lipolytic activity of adherent adipocytes across scaffold conditions, suggesting that the increase in total DNA for NaOH-degummed scaffolds may be partly a result of increased attachment of non-adipocyte cell types. Additionally, glycerol content was similar to observed levels for adipose tissue seeded into HFIP-based silk scaffolds in prior research [44], indicating expected levels of adipocyte functioning in the more environmentally friendly scaffold environment. Findings collectively indicate that the more environmentally friendly scaffolds provide an equally biocompatible environment for stem cell and soft tissue applications compared to aqueous-based scaffolds produced following the well-established Na_2_CO_3_ degumming approach, with increased cellular attachment over the current gold standard that correlates to the higher porosity of NaOH-degummed scaffolds (as indicated earlier in Figure 3D).

### 3.5. Limitations

After degumming with both the green NaOH-based approach and the established Na_2_CO_3_ degumming method, lithium bromide was used to dissolve fibroin to produce aqueous solutions. The prior literature describes several environmental concerns with the established LiBr-based dissolution method, including pollution to the water, air, and soil as a result of lithium mining; the requirement of high LiBr salt concentrations as well as large amounts of water consumption; the detrimental effects to human and aquatic animal health; and high energy usage to continuously heat LiBr solutions to 60 °C for silk dissolution [25,65,66]. A few green alternative solvent systems have been proposed, including NaOH and deep eutectic solvents [13,25,67], but were not used in this study due either to the complexity of methodology or difficulty in replicating results. As such, aqueous silk solutions prepared following NaOH-based degumming are not a “fully” green alternative, and thus there is still room to make a bottom-up green processing approach that includes a green dissolution method as well. However, the experimental method for preparing aqueous silk solutions developed in this study still managed to mitigate some of the environmental concerns associated with the current gold standard methodology.

## 4. Conclusions

This study optimized an approach for degumming *Bombyx mori* silk via NaOH and paired it with an aqueous-based silk fibroin scaffold fabrication process to create a viable and more environmentally friendly biomaterial platform, with morphology, protein structure, mechanical properties, and degradation kinetics that are comparable to silk scaffolds produced using non-sustainable degumming scaffold fabrication methods. In addition to being a more environmentally friendly alternative compared to well-established processing steps, results indicate that aqueous-based silk scaffolds produced following NaOH degumming have a higher porosity which was correlated with a higher degree of cell attachment (DNA content) for adipose tissue. The more environmentally friendly silk scaffolds also demonstrated high viability with hASCs, as well as healthy unilocular adipocyte morphology and function, with similar results to scaffolds produced via the well-established Na_2_CO_3_ degumming method. These findings indicate that the more environmentally friendly silk fibroin scaffolds are well suited for use in adipose tissue applications (given the similarity in compressive modulus to that of adipose tissue), as well as other soft tissue types with similar properties.

## Figures and Tables

**Table 1:** Characteristic silk I and silk II absorbance peak ranges on IR spectra for functional amides in silk fibroin [46].

Functional Amide	Silk I Range (cm^−1^)	Silk II Range (cm^−1^)
Amide I ^1^	1648–1654	1610–1630
Amide II ^2^	1535–1542	1510–1520

^1^ primarily due to C=O stretching vibration. ^2^ primarily due to C–N stretching and N–H bending vibrations.

**Table 2:** Absorbance band wavenumbers indicative of characteristic differences between silk fibroin and silk sericin proteins, resulting from differences in content of specific amino acids [45,46].

Absorbance Band (cm^−1^)	Primary Bond Vibration	Indicated Protein
1070	Serine side chain ^1^	Sericin
1165	Tyrosine side chain	Fibroin
1400	Aspartic acid side chain ^2^	Sericin
1445	Alanine side chain	Fibroin
1625	Amide I ^3^	Fibroin
1650	Amide I ^4^	Sericin

^1^ alkyl hydroxyl-containing amino acid side chain. ^2^ carboxylic acid-containing amino acid side chain. ^3^ C=O stretching, band wavenumber found in silk fibroin in silk II form. ^4^ C=O stretching, band wavenumber found in silk sericin.

## Data Availability

Data are available from the corresponding author via email.

## References

[1] Vepari C., Kaplan D.L. (2007). Silk as a biomaterial. Prog. Polym. Sci..

[2] Cao T.T., Zhang Y.Q. (2016). Processing and characterization of silk sericin from Bombyx mori and its application in biomaterials and biomedicines. Mater. Sci. Eng. C Mater. Biol. Appl..

[3] Shao Z., Vollrath F. (2002). Surprising strength of silkworm silk. Nature.

[4] Koh L.-D., Cheng Y., Teng C.-P., Khin Y.-W., Loh X.-J., Tee S.-Y., Low M., Ye E., Yu H.-D., Zhang Y.-W. (2015). Structures, mechanical properties and applications of silk fibroin materials. Prog. Polym. Sci..

[5] Kundu B., Kurland N.E., Bano S., Patra C., Engel F.B., Yadavalli V.K., Kundu S.C. (2014). Silk proteins for biomedical applications: Bioengineering perspectives. Prog. Polym. Sci..

[6] Hu X., Shmelev K., Sun L., Gil E.-S., Park S.-H., Cebe P., Kaplan D.L. (2011). Regulation of Silk Material Structure by Temperature-Controlled Water Vapor Annealing. Biomacromolecules.

[7] Diab T., Pritchard E.M., Uhrig B.A., Boerckel J.D., Kaplan D.L., Guldberg R.E. (2012). A silk hydrogel-based delivery system of bone morphogenic protein for the treatment of large bone defects. J. Mech. Behav. Biomed. Mater..

[8] Chao P.-H.G., Yodmuang S., Wang X., Sun L., Kaplan D.L., Vunjak-Novakovic G. (2010). Silk hydrogel for cartilage tissue engineering. J. Biomed. Mater. Res. Part B Appl. Biomater..

[9] Cao Y., Wang B. (2009). Biodegradation of Silk Biomaterials. Int. J. Mol. Sci..

[10] Rockwood D.N., Preda R.C., Yucel T., Wang X., Lovett M.L., Kaplan D.L. (2011). Materials fabrication from *Bombyx mori* silk fibroin. Nat. Protoc..

[11] Gellynck K., Verdonk P.C.M., Van Nimmen E., Almqvist K.F., Gheysens T., Schoukens G., Van Langenhove L., Kiekens P., Mertens J., Verbruggen G. (2008). Silkworm and spider silk scaffolds for chondrocyte support. J. Mater. Sci. Mater. Med..

[12] Xu J., Dong Q., Yu Y., Niu B., Ji D., Li M., Huang Y., Chen X., Tan A. (2018). Mass spider silk production through targeted gene replacement in Bombyx mori. Proc. Natl. Acad. Sci. USA.

[13] Kundu B., Kurland N.E., Yadavalli V.K., Kundu S.C. (2014). Isolation and processing of silk proteins for biomedical applications. Int. J. Biol. Macromol..

[14] Kwak H.W., Ju J.E., Shin M., Holland C., Lee K.H. (2017). Sericin Promotes Fibroin Silk I Stabilization Across a Phase-Separation. Biomacromolecules.

[15] Mahmoodi N.M., Arami M., Mazaheri F., Rahimi S. (2010). Degradation of sericin (degumming) of Persian silk by ultrasound and enzymes as a cleaner and environmentally friendly process. J. Clean. Prod..

[16] Yang Y., Chen X., Ding F., Zhang P., Liu J., Gu X. (2007). Biocompatibility evaluation of silk fibroin with peripheral nerve tissues and cells in vitro. Biomaterials.

[17] Boonrungsiman S., Thongtham N., Suwantong O., Wutikhun T., Soykeabkaew N., Nimmannit U. (2018). An improvement of silk-based scaffold properties using collagen type I for skin tissue engineering applications. Polym. Bull..

[18] Talukdar S., Nguyen Q.T., Chen A.C., Sah R.L., Kundu S.C. (2011). Effect of initial cell seeding density on 3D-engineered silk fibroin scaffolds for articular cartilage tissue engineering. Biomaterials.

[19] Kim H.J., Kim M.K., Lee K.H., Nho S.K., Han M.S., Um I.C. (2017). Effect of degumming methods on structural characteristics and properties of regenerated silk. Int. J. Biol. Macromol..

[20] Kundu B., Rajkhowa R., Kundu S.C., Wang X. (2013). Silk fibroin biomaterials for tissue regenerations. Adv. Drug Deliv. Rev..

[21] Lamboni L., Gauthier M., Yang G., Wang Q. (2015). Silk sericin: A versatile material for tissue engineering and drug delivery. Biotechnol. Adv..

[22] Alfian M., Purwanto W.W. (2019). Multi-objective optimization of green urea production. Energy Sci. Eng..

[23] Lin Y., Xu D., Zhao X. (2021). Properties and Hydration Mechanism of Soda Residue-Activated Ground Granulated Blast Furnace Slag Cementitious Materials. Materials.

[24] Wang H.-Y., Zhang Y.-Q. (2013). Effect of regeneration of liquid silk fibroin on its structure and characterization. Soft Matter.

[25] Debari M.K., King C.I., Altgold T.A., Abbott R.D. (2021). Silk Fibroin as a Green Material. ACS Biomater. Sci. Eng..

[26] Rajan M.K., Balakrishnan A., Jayaraman K. (1992). Development of an antibody against a 170-kDa fragment of fibroin isolated from cocoon fibres of *Bombyx mori*. J. Biochem. Biophys. Methods.

[27] Du F., Warsinger D.M., Urmi T.I., Thiel G.P., Kumar A., Lienhard V.J. (2018). Sodium Hydroxide Production from Seawater Desalination Brine: Process Design and Energy Efficiency. Environ. Sci. Technol..

[28] Kim H., Kim H., Matsumoto A., Chin I.-J., Jin H.-J., Kaplan D. (2005). Processing Windows for Forming Silk Fibroin Biomaterials into a 3D Porous Matrix. Aust. J. Chem..

[29] Burke K.A., Roberts D.C., Kaplan D.L. (2016). Silk Fibroin Aqueous-Based Adhesives Inspired by Mussel Adhesive Proteins. Biomacromolecules.

[30] Park S.-H., Gil E.S., Shi H., Kim H.J., Lee K., Kaplan D.L. (2010). Relationships Between Degradability of Silk Scaffolds and Osteogenesis. Biomaterials.

[31] Mauney J.R., Nguyen T., Gillen K., Kirker-Head C., Gimble J.M., Kaplan D.L. (2007). Engineering adipose-like tissue in vitro and in vivo utilizing human bone marrow and adipose-derived mesenchymal stem cells with silk fibroin 3D scaffolds. Biomaterials.

[32] Abbott R.D., Raja W.K., Wang R.Y., Stinson J.A., Glettig D.L., Burke K.A., Kaplan D.L. (2015). Long term perfusion system supporting adipogenesis. Methods.

[33] Bellas E., Marra K., Kaplan D.L.P. (2013). Sustainable Three-Dimensional Tissue Model of Human Adipose Tissue. Tissue Eng. Part C Methods.

[34] Choi J.H., Gimble J.M., Lee K., Marra K.G., Rubin J.P., Yoo J.J., Vunjak-Novakovic G., Kaplan D.L. (2010). Adipose tissue engineering for soft tissue regeneration. Tissue Eng. Part B Rev..

[35] Choi J.H., Gimble J.M., Vunjak-Novakovic G., Kaplan D.L. (2010). Effects of hyperinsulinemia on lipolytic function of three-dimensional adipocyte/endothelial co-cultures. Tissue Eng. Part C Methods.

[36] Kang J.H., Gimble J.M., Kaplan D.L. (2009). In vitro 3D model for human vascularized adipose tissue. Tissue Eng. Part A.

[37] Ward A., Quinn K.P., Bellas E., Georgakoudi I., Kaplan D.L. (2013). Noninvasive Metabolic Imaging of Engineered 3D Human Adipose Tissue in a Perfusion Bioreactor. PLoS ONE.

[38] Choi J.H., Bellas E., Vunjak-Novakovic G., Kaplan D.L. (2011). Adipogenic differentiation of human adipose-derived stem cells on 3D silk scaffolds. Methods Mol. Biol..

[39] DeBari M.K., Niu X., Scott J.V., Griffin M.D., Pereira S.R., Cook K.E., He B., Abbott R.D. (2021). Therapeutic Ultrasound Triggered Silk Fibroin Scaffold Degradation. Adv. Healthc. Mater..

[40] Bender R., McCarthy M., Brown T., Bukowska J., Smith S., Abbott R.D., Kaplan D.L., Williams C., Wade J.W., Alarcon A. (2020). Human Adipose Derived Cells in Two- and Three-Dimensional Cultures: Functional Validation of an In Vitro Fat Construct. Stem Cells Int..

[41] Vidal S.E.L., Tamamoto K.A., Nguyen H., Abbott R.D., Cairns D.M., Kaplan D.L. (2019). 3D biomaterial matrix to support long term, full thickness, immuno-competent human skin equivalents with nervous system components. Biomaterials.

[42] Abbott R.D., Borowsky F.E., Alonzo C.A., Zieba A., Georgakoudi I., Kaplan D.L. (2018). Variability in responses observed in human white adipose tissue models. J. Tissue Eng. Regen. Med..

[43] Wang R.Y., Abbott R.D., Zieba A., Borowsky F.E., Kaplan D.L. (2017). Development of a Three-Dimensional Adipose Tissue Model for Studying Embryonic Exposures to Obesogenic Chemicals. Ann. Biomed. Eng..

[44] Abbott R.D., Wang R.Y., Reagan M.R., Chen Y., Borowsky F.E., Zieba A., Marra K.G., Rubin J.P., Ghobrial I.M., Kaplan D.L. (2016). The Use of Silk as a Scaffold for Mature, Sustainable Unilocular Adipose 3D Tissue Engineered Systems. Adv. Healthc. Mater..

[45] Zhang X., Wyeth P. (2010). Using FTIR spectroscopy to detect sericin on historic silk. Sci. China Chem..

[46] Chatterley A.S., Laity P., Holland C., Weidner T., Woutersen S., Giubertoni G. (2022). Broadband Multidimensional Spectroscopy Identifies the Amide II Vibrations in Silkworm Films. Molecules.

[47] Li M., Ogiso M., Minoura N. (2003). Enzymatic degradation behavior of porous silk fibroin sheets. Biomaterials.

[48] Brown J., Lu C.-L., Coburn J., Kaplan D.L. (2015). Impact of silk biomaterial structure on proteolysis. Acta Biomater..

[49] Numata K., Cebe P., Kaplan D.L. (2010). Mechanism of enzymatic degradation of beta-sheet crystals. Biomaterials.

[50] Wray L.S., Hu X., Gallego J., Georgakoudi I., Omenetto F.G., Schmidt D., Kaplan D.L. (2011). Effect of processing on silk-based biomaterials: Reproducibility and biocompatibility. J. Biomed. Mater. Res. Part B Appl. Biomater..

[51] Asakura T., Ashida J., Yamane T., Kameda T., Nakazawa Y., Ohgo K., Komatsu K. (2001). A repeated β-turn structure in Poly(Ala-Gly) as a model for silk I of Bombyx mori silk fibroin studied with two-dimensional spin-diffusion NMR under off magic angle spinning and rotational echo double resonance11Edited by M. F. Summers. J. Mol. Biol..

[52] Debari M.K., Abbott R.D. (2019). Microscopic considerations for optimizing silk biomaterials. WIREs Nanomed. Nanobiotech..

[53] Zhao H.-P., Feng X.-Q., Shi H.-J. (2007). Variability in mechanical properties of Bombyx mori silk. Mater. Sci. Eng. C.

[54] Liebschner M., Bucklen B., Wettergreen M. (2005). Mechanical Aspects of Tissue Engineering. Semin. Plast. Surg..

[55] Griffin M., Premakumar Y., Seifalian A., Butler P.E., Szarko M. (2016). Biomechanical Characterization of Human Soft Tissues Using Indentation and Tensile Testing. J. Vis. Exp..

[56] Chan B.P., Leong K.W. (2008). Scaffolding in tissue engineering: General approaches and tissue-specific considerations. Eur. Spine J..

[57] Sharifi M., Farahani M.K., Salehi M., Atashi A., Alizadeh M., Kheradmandi R., Molzemi S. (2023). Exploring the Physicochemical, Electroactive, and Biodelivery Properties of Metal Nanoparticles on Peripheral Nerve Regeneration. ACS Biomater. Sci. Eng..

[58] Sun Z., Lee S.-H., Gepner B.D., Rigby J., Hallman J.J., Kerrigan J.R. (2021). Comparison of porcine and human adipose tissue loading responses under dynamic compression and shear: A pilot study. J. Mech. Behav. Biomed. Mater..

[59] Jain S., Yassin M.A., Fuoco T., Mohamed-Ahmed S., Vindenes H., Mustafa K., Finne-Wistrand A. (2021). Understanding of how the properties of medical grade lactide based copolymer scaffolds influence adipose tissue regeneration: Sterilization and a systematic in vitro assessment. Mater. Sci. Eng. C.

[60] Franz S., Rammelt S., Scharnweber D., Simon J.C. (2011). Immune responses to implants—A review of the implications for the design of immunomodulatory biomaterials. Biomaterials.

[61] Gonzalez A.C.D.O., Costa T.F., Andrade Z.D.A., Medrado A.R.A.P. (2016). Wound healing—A literature review. An. Bras. Dermatol..

[62] Shao M., Bigham A., Yousefiasl S., Yiu C.K.Y., Girish Y.R., Ghomi M., Sharifi E., Sezen S., Nazarzadeh Zare E., Zarrabi A. (2023). Recapitulating Antioxidant and Antibacterial Compounds into a Package for Tissue Regeneration: Dual Function Materials with Synergistic Effect. Small.

[63] Abbott R.D., Kimmerling E.P., Cairns D.M., Kaplan D.L. (2016). Silk as a Biomaterial to Support Long-Term Three-Dimensional Tissue Cultures. ACS Appl. Mater. Interfaces.

[64] Georgakoudi I., Tsai I., Greiner C., Wong C., Defelice J., Kaplan D. (2007). Intrinsic fluorescence changes associated with the conformational state of silk fibroin in biomaterial matrices. Opt. Express.

[65] Wanger T.C. (2011). The Lithium future-resources, recycling, and the environment. Conserv. Lett..

[66] Shen T., Wang T., Cheng G., Huang L., Chen L., Wu D. (2018). Dissolution behavior of silk fibroin in a low concentration CaCl2-methanol solvent: From morphology to nanostructure. Int. J. Biol. Macromol..

[67] Hu Y., Liu L., Yu J., Wang Z., Fan Y. (2020). Preparation of Natural Multicompatible Silk Nanofibers by Green Deep Eutectic Solvent Treatment. ACS Sustain. Chem. Eng..

